# Aerobic training modulates T cell activation in elderly women with knee osteoarthritis

**DOI:** 10.1590/1414-431X20165181

**Published:** 2016-11-03

**Authors:** W.F. Gomes, A.C.R. Lacerda, G.E.A. Brito-Melo, S.F. Fonseca, E. Rocha-Vieira, A.A.O. Leopoldino, M.R. Amorim, V.A. Mendonça

**Affiliations:** 1 Centro Integrado de Pós-Graduação e Pesquisa em Saúde (CIPq-Saúde), Universidade Federal dos Vales do Jequitinhonha e Mucuri, Diamantina, MG, Brasil; 2 Programa Multicêntrico de Pós Graduação em Ciências Fisiológicas (PMPGCF), Sociedade Brasileira de Fisiologia (SBFis), Brasil; 3 Departamento de Fisioterapia e Terapia Ocupacional, Escola de Educação Física, Universidade Federal de Minas Gerais, Belo Horizonte, MG, Brasil; 4 Departamento de Fisiologia, Faculdade de Medicina de Ribeirão Preto, Universidade de São Paulo, Ribeirão Preto, SP, Brasil; 5 Programa de Pós-Graduação em Reabilitação e Desempenho Funcional (PPGReab), Departamento de Fisioterapia, Universidade Federal dos Vales do Jequitinhonha e Mucuri, Diamantina, MG, Brasil

**Keywords:** Osteoarthritis, Elderly, Exercise, CD4-positive T-lymphocytes, CD8-positive T-lymphocytes

## Abstract

Osteoarthritis of the knee (kOA) is a disease that mainly affects the elderly and can lead to major physical and functional limitations. However, the specific effects of walking, particularly on the immune system, are unknown. Therefore, this study aimed to analyze the effect of 12 weeks of walking (3×/week) on the leukocyte profile and quality of life (QL) of elderly women with kOA. Sixteen women (age: 67±4 years, body mass index: 28.07±4.16 kg/m^2^) participated in a walking program. The variables were assessed before and after 12 weeks of training with a progressively longer duration (30–55 min) and higher intensity (72–82% of HRmax determined using a graded incremental treadmill test). The QL was assessed using the Medical Outcomes Study 36-Item Short Form Health Survey (SF-36), and blood samples were collected for analysis with a cell counter and the San Fac flow cytometer. Walking training resulted in a 47% enhancement of the self-reported QL (P<0.05) and a 21% increase in the VO_2_max (P<0.0001) in elderly women with kOA. Furthermore, there was a reduction in CD4+ cells (pre=46.59±7%, post=44.58±9%, P=0.0189) and a higher fluorescence intensity for CD18+CD4+ (pre=45.30±10, post=64.27±33, P=0.0256) and CD18^+^CD8^+^ (pre=64.2±27, post=85.02±35, P=0.0130). In conclusion, the walking program stimulated leukocyte production, which may be related to the immunomodulatory effect of exercise. Walking also led to improvements in the QL and physical performance in elderly women with kOA.

## Introduction

Aging is an issue that demands increasing attention in the field of elderly care, specifically in women with knee osteoarthritis (kOA) (1,2). Previous studies indicate that the aging process is associated with an underlying chronic inflammatory state. This state is characterized by an approximately two- to four-fold increase in the plasma levels of inflammatory cytokines, as well as cell-specific activation and increased cell migration (3,4). The mechanisms related to the increased production and release of cytokines, and the activation and migration of cells involved in the inflammatory process remain to be elucidated. There are several factors that appear to be involved in inflammation, including the presence of chronic disease, decreased production of sex steroids, psychosocial factors and increased adipose tissue (4). The inflammatory changes associated with aging and kOA play an important role in the protein catabolism of muscle fibers, resulting in sarcopenia and, thus, functional changes that can be controlled by exercise (5).

Osteoarthritis is a chronic degenerative disease in which the knee is the most affected weight-bearing joint. This disease affects the main structures of the joint complex and may cause local pain and severe functional limitations, resulting in a declining quality of life in the elderly (2,6,7). kOA was initially thought to be a noninflammatory disease, but the roles of synovitis, bone and muscle alterations in kOA have demonstrated the influence of inflammation and have shown that kOA is a disease not only of the cartilage but also of the joints, with immunological systemic consequences (3,7,8). Synovial fluid within the joint is considered to be the best fluid for analyzing immune-inflammatory factors in kOA (9). However, because of the technical difficulty and risk, human studies have examined the behavior of these immune-inflammatory factors in the blood (10,11). Furthermore, blood analysis also allows to assess long-term therapeutic results (12) and has been widely used in studies with kOA (13–16).

Current international guidelines recommend therapeutic exercise (land- or water-based) for kOA as "core" and effective management, given its beneficial effects, ease of application, few adverse effects, and relatively low cost (17). Regular walking is often recommended for the elderly because of the facility of implementation and the obtained results described in the literature (18). Studies have shown that regular exercise of moderate intensity positively affects the immune system because of the associated anti-inflammatory effect (19–21). Moreover, exercise induces immunomodulatory effects, such as changes in the number and function of peripheral blood cells (neutrophils, B, T, NK and monocytes), and it influences the trafficking of cells, such as CD8^+^ lymphocytes, between the blood and target tissues in healthy individuals (22).

The symptoms of kOA have a negative impact on health-related parameters in the elderly population, such as physical and functional performance and vitality, as well as social, mental and emotional characteristics (23).

According to the current literature, low intensity exercise can modulate the inflammatory response in individuals with chronic diseases (11,19). According to Gomes et al. (10), acute and chronic aerobic exercise resulted in a change in sTNFR1 and sTNFR2 levels that correlated with functional improvements in elderly women with kOA.

Therefore, considering the increase in the number of individuals with kOA and the need to understand the effects of exercise on the immune system in this population, it is clinically relevant to evaluate the effect of aerobic training (12 weeks, three times per week) on the quality of life in elderly women with kOA. Similarly, the balance analysis of circulating leukocytes and immunological parameters related to activation and migration of subpopulations of T lymphocytes is also needed.

It is believed that the anti-inflammatory and immunomodulatory effects of aerobic exercise training are mediated by changes in the leukocyte profile in the peripheral blood of elderly women with kOA. Furthermore, the aerobic exercise training would have a positive influence in activating immune markers of activated lymphocytes and controlling local inflammation. As a consequence, it would improve the perceived quality of life in this population.

## Material and Methods

### Ethical statement

This is a quasi-experimental study in which the dependent variables were assessed before and after training. This study was conducted in accordance with the ethical principles for research involving humans (expressed in the Declaration of Helsinki) and received approval from the Ethics Committee of the Universidade Federal dos Vales do Jequitinhonha e Mucuri (protocol No. 101/09). All participants gave their written informed consent to participate.

### Subjects

The study subjects consisted of elderly women from the general community of Diamantina, MG, Brazil, with clinically and radiographically diagnosed kOA. All patients met the following inclusion criteria: 1) aged 65 years and over, 2) OA diagnosis in at least one knee based on the clinical and radiographic criteria of the American College of Rheumatology with radiographic classification, 3) no prior surgical procedure in the lower limbs, 4) no history of recent trauma to the knees, 5) no use of mobility aids (canes, crutches, and walkers), 6) no history of physical therapy or any other procedure for rehabilitation in the last 3 months as well as not exercising regularly, 7) met the clinical conditions and cognitive requirements for the exercises, 8) completion of the Mini-Mental State Examination (MMSE), 9) no use of any glucocorticoids for at least 2 months, and 10) no use of beta-blocker drugs. Patients unable to finish the test run used to determine the level of physical capability and which was required to complete this study were removed.

### Procedures

Demographic data were collected after the inclusion criteria had been met. The patients were subjected to the maximal exercise test on a treadmill to determine their aerobic performance for the required training in the study. One week later and 24 h before the beginning of the training program, the quality of life of the subjects was assessed using the Short Form-36 (SF-36) generic instrument. Additionally, 6 mL of venous peripheral blood was collected from each subject, with heparin as an anticoagulant, 24 h after 12 weeks of a walking-training program; the volunteers were again subjected to the maximal exercise test on a treadmill to determine aerobic performance. One week later, the quality of life of the subjects followed by blood sample collection were repeated. It is important to emphasize that all experimental procedures were performed at the same time of the day, always in the morning.

### Radiographic evaluation

To ensure that the participants had kOA and to reliably standardize the samples, a radiological evaluation was performed on all of the volunteers. Anteroposterior, oblique, and lateral images of the most affected knee were taken in the standing position. A radiological classification was made in accordance with the Kellgren-Lawrence (24).

### Training

Training consisted of walking with a progressive increase in the exercise load. This was always performed in the afternoon three times per week (Mondays, Wednesdays and Fridays with weekends off), for 12 straight weeks. The sessions were controlled in terms of the progression of time and intensity. Walking intensity was monitored based on the target heart rate of each volunteer, which was monitored by a heart rate monitor (model F4, Polar, Brazil). During each training session, the participants’ heart rate was checked by a supervisor and recorded every 3 min to ensure that heart rate was maintained within the range previously determined (target heart rate±5 bpm). The exercise consisted of three distinct stages: warm up (5 min), aerobic exercise–walking (initially 30 min, 70% HR_max_), and cool down (5 min). The volume of aerobic exercise was individually prescribed and changed gradually in 5-min increments every 2 weeks (30 min in the first week and up to 55 min in the last week) with a similar progression for target heart rate training (72% HR_max_ from 1st to 3rd week, 77% HR_max_ from 4th to 7th week, and 82% HR_max_ from 8th to 12th week) (Table 1).

**Table:**
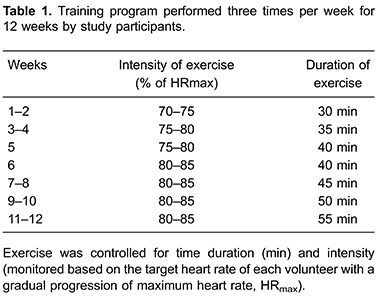

### Aerobic performance

To determine the aerobic performance before and after the training program, a progressive exercise test was performed until fatigue on a treadmill (classic mode, Inbramed, Brazil). Heart rate was recorded every 30 s during the test using a heart rate monitor, and Borg's rating of perceived exertion (RPE) scale was administered every 3 min during the test (25,26). The test was stopped, and maximal exertion was said to be reached if a score of more than 18 on the Borg scale and/or volitional fatigue was reported (25,26). In addition, the test was discontinued in the event of dizziness, nausea, blurred vision, dyspnea, chest pain, elevated diastolic blood pressure to 120 mmHg, sustained drop in systolic blood pressure (SBP) or marked elevation of SBP to 260 mmHg. The maximum oxygen consumption (VO_2max_) was calculated based on the slope grade (G) and speed (S) during the last stage of the test completed by the subject according to the following equation: VO_2_ = (0.1×S) + (1.8×S×G) + 3.5 (26).

### Quality of life

To determine the perception of quality of life before and after the training program, the Short Form Health Survey (SF-36) was applied. The SF-36 is a multidimensional test that is composed of 36 items divided into physical and emotional components, and evaluates eight of the main domains related to health. In each domain, the punctuation varies from 0 to 100. A higher score indicates a better quality of life. The SF-36 has been validated and adapted for the Brazilian culture (27).

### Cell parameters

Cell immunophenotyping was performed to evaluate the biological material before and after the training program. The results are reported as percentage values for the cell balance analysis, and the mean fluorescence intensity (MFI) was used to analyze the relative density of receptor expression on the cell surface.

**Figure 1:**
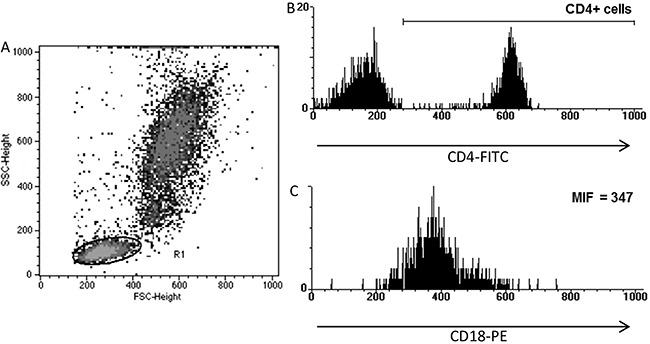
Flow cytometry data analysis. Lymphocytes were gated based on forward- and side-scattered light (R1, in *A*). After selection of the population of interest (*B*), the density of CD18 expression on the cell surface was evaluated by mean fluorescence intensity (*C*). MIF: mean fluorescence intensity.

Fifty microliters of peripheral blood was incubated with fluorescently conjugated monoclonal antibodies against cell surface markers, including CD3-FITC (UCTH-1, Immunotech, USA), CD4-PeCy5 (RPA-T4, BD Bioscience, USA) and CD8-PeCy5 (RPA-T4, BD Bioscience), for the analysis of the subsets of T lymphocytes, CD19 (J4.119, Immunotech) for the analysis of B lymphocytes, and CD56-PE (N901, Immunotech) and CD16-PeCy5 (3G8, Immunotech) for the analysis of NK and NKT cells. CD28-PE (LS198-4-3, Immunotech) and HLA-DR-PE (TU36, Pharmingen, USA) were also evaluated because of their role in cell activation, and CD18-PE (7E4, Immunotech), an integrin, was assessed because of its involvement in the migration process. Rat anti-mouse IgG1-FITC (A85-1, BD-Pharmingen, USA), rat anti-mouse IgG2a+b-PE (X57, BD-Pharmingen), and rat anti-mouse IgG2a+b-PerCy5 (X57, BD-Pharmingen) were used as isotype controls. Then, the erythrocytes were lysed (FACSLyse solution, BD, USA), and the remaining cells were washed twice using saline solution. The frequency of CD3^+^CD4^+^, CD4^+^ CD28^+^, CD3^+^CD8^+^, CD8^+^CD28^+^, CD3^-^CD19^+^, CD3^+^ CD56^+^CD16^+^ and CD3^-^CD56^+^CD16^+^ lymphocytes and the density of CD18 expression by lymphocytes was evaluated using the FACScan (BD) flow cytometer equipped with a blue argon laser (488 nm) and the following filters: 530/30 nm (FL1=green fluorescence) and 586/42 (FL2= orange fluorescence) band-pass filters, as well as the 650/LP (FL3=red fluorescence) long-pass filter. Ten thousand events were acquired, and data were analyzed using the Cell Quest software (BD). The frequencies of the cellular subpopulations were determined in the lymphocyte gate, according to forward and side scatter parameters (R1, Figure 1A). For the analysis of cell marker expression by lymphocytes, after selection of CD4- or CD8-positive cells (Figure 1B), the mean fluorescence intensity (MFI) of the receptor of interest was evaluated (Figure 1C).

### Statistical analysis

The sample size was determined based on a pilot study with 4 subjects (28). From the data for the variable of interest, the sample size was calculated considering an alpha of 5% and a power of 80% for a sample composed of 16 subjects. Descriptive analysis and normality tests (Shapiro-Wilk) were performed using the statistical package GraphPad Prism (GraphPad Software Inc., USA). Because all data were normally distributed, statistical analysis was performed using paired Student's *t*-test. The results were considered to be statistically significant at a level of 5%.

## Results

**Figure 2:**
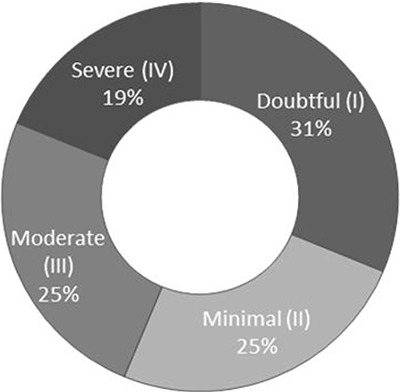
Knee osteoarthritis x-ray classification of the 16 volunteers of this study according to Kellgren and Lawrence (24) I to V degrees.

The samples were evaluated from 16 volunteers with clinical and radiographic diagnosis of kOA (Figure 2). All subjects (mean age: 67±4 years; mean body mass index: 28±4 kg/m^2^) completed the 12-week training program with an overall adherence of 94%. The training program did not change the mean body mass index of participants (27±2 kg/m^2^).

Physical performance was determined using the values obtained for maximum oxygen consumption (VO_2max_) during the progressive tests to fatigue performed on a treadmill before and after training. The VO_2max_ increased by an average of 21% with training (pre=28±5 mL·kg^-1^·min^-1^, post=34±5 mL·kg^-1^·min^-1^ and P<0.0001).

**Figure 3:**
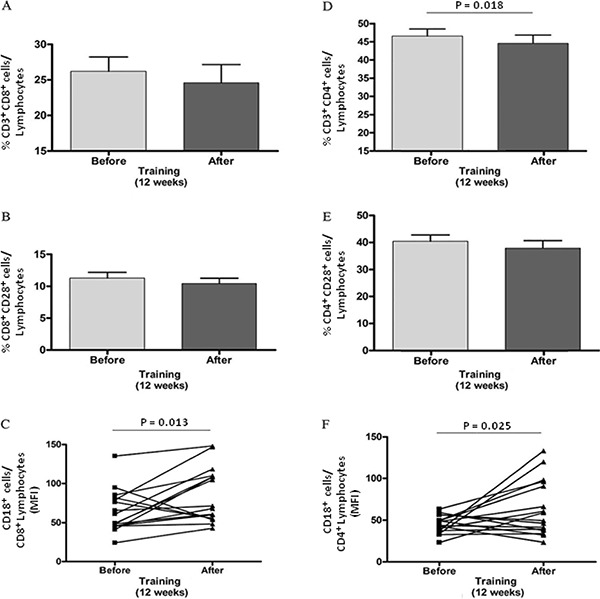
Phenotypic analysis of circulating leukocytes in peripheral blood of patients. Mean value of the percentage of subpopulations of TCD8+ cells (*A*) and TCD4+ cells-B lymphocytes (*D*), and the state of cell activation in CD8+ and CD4+ T cells by analysis of co-stimulatory molecule CD28 (*B* and *E*, respectively). CD18 expression by lymphocytes considering individual values before and after training is shown in *C* (CD8+ T cells) and *F* (CD4+ T cells). MFI: mean fluorescence intensity. Statistical analysis was performed with Student's *t*-test.

In addition to improving the aerobic performance, the training affected the distribution of certain leukocyte populations and subpopulations. The percentage of the circulating lymphocytes increased after training and the neutrophil percentage was reduced (Table 2). Although the lymphocyte percentage was reduced after 12 weeks of training, there was no change in the percentage of CD8^+^ cells (Figure 3A) (pre=26±8%, post=24±10% and P=0.1604) or CD8^+^CD28^+^ (Figure 3B) compared with values obtained before the intervention (pre=11±3%, post=10±3% and P=0.1227). However, regarding the mean fluorescence intensity, there was an increase in the expression of CD18 by CD8^+^ cells (Figure 3C) (pre=64±27, post=85±35 and P=0.0130).

**Table:**
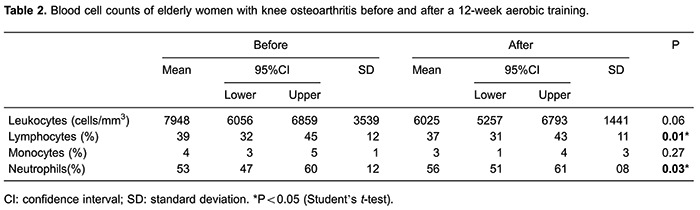

In contrast to the results for CD8^+^ cells, the percentage of CD4^+^ cells after training was lower than that observed before the intervention (Figure 3D) (pre=46±7%, post=44±9% and P=0,0189). However, similar to the results for CD8^+^ cells, the percentage of CD4^+^CD28^+^ lymphocytes did not change with training (Figure 3E) (pre=40±9, post=38±11 and P=0.0505). The expression of CD18 by CD4^+^ cells after training was higher compared to pre-training (Figure 3F) (pre=45±10, post=64±33 and P=0.0256).

**Figure 4:**
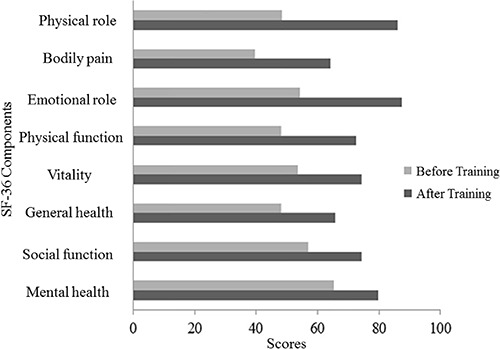
Quality of life perception scores using the Short Form Health Survey (SF-36), before and after training. In each domain, the score varies from 0 to 100.

The self-perceived quality of life was positively enhanced by 47% at the end of the 12-week intervention (Figure 4), indicating improvement in all of the 4 physical components of the SF-36: physical function (72±2, P<0.01, 50% increase), physical role (86±3, P<0.01, 77% increase), bodily pain (64±2, P<0.01, 62% increase), and general health (66±2, P<0.01, 36% increase). The mental components also showed improvement: social function (74±4, P<0.05, 30% increase), emotional role (87±6, P<0.05, 61% increase), mental health (80±3; P<0.05, 22% increase) and vitality (74±2, P<0.01, 39% increase).

## Discussion

The results of this study demonstrate that the proposed walking training increased aerobic performance, improved the self-rated quality of life and mobilized a distinct immune response in elderly women with kOA. Specifically, we evaluated the change in the average percentages of cell populations/subpopulations that are primarily associated with cell migration, after training.

It has been suggested that the clinical course of osteoarthritis is influenced by mechanisms related to the effector function of T lymphocytes (3). T lymphocytes are sub-classified into CD4^+^ and CD8^+^ cells and are affected by various factors such as the microenvironment. Stimulation by soluble and membrane-bound factors may direct the immune response mechanisms linked to the maintenance or resolution of the inflammatory response.

Given this complex network of interactions between cells and stimulation, T lymphocytes respond in diverse and sometimes antagonistic ways. For example, T lymphocytes, such as Th1 (CD3^+^CD4^+^TNF^+^), Th17 (CD3^+^CD4^+^IL-17^+^) and cytotoxic T lymphocytes (CD3^+^CD8^+^), can intensify the inflammatory response. However, they can also reduce the inflammatory response, as in the case of Treg lymphocytes (CD4^+^CD25^high^FOXP3^+^). The mechanisms related to how and when these elements function, particularly in the immune system of older individuals, are the subjects of ongoing investigations (29).

Clinical studies have shown that the immune system responds to increased physical activity. The effects of intense exercise on the immune response differ from those observed during moderate-intensity exercise; individuals participating in frequent high-intensity exercise appear to be more immunocompromised than those who perform exercise of moderate physical intensity. In this context, it has been proposed that the intensity of exercise influences the subpopulations of Th17 and Treg cells. The Th17 T-helper lymphocytes are CD4^+^ T lymphocytes that produce IL-17, a cytokine that plays a crucial role in the allergic inflammatory process, and are known as a potent pro-inflammatory cell type in autoimmune chronic-degenerative diseases (19). Treg are differentiated and actively involved in the control of the peripheral immune response. Treg cells originate from precursor CD4^+^CD25^-^ lymphocytes, which when stimulated by TGF-β and IL-2, signal the expression of FOXP3 to maintain their regulatory function. The involvement of CD8^+^ T lymphocytes (CD3^+^CD8^+^ CD45RC^low^) in immunosuppressive mechanisms has also been reported; however, the regulatory mechanisms of this cell population remain unknown (30). Considering the role of T lymphocyte subsets as suppressors in the chronic inflammatory response, we sought to verify whether phenotypical changes in T-CD4^+^ and T-CD8^+^ lymphocytes could be associated with clinical improvement of patients with kOA. Migration-related parameters (CD18), as well as markers of cell activation (CD28) that provide relevant information about a probable immunomodulation of the inflammatory response, were chosen for this purpose.

The present study demonstrated that physical exercise in elderly individuals with kOA promoted a significant improvement in function and quality of life. A comparison between before and after training demonstrated a decrease in peripheral blood lymphocytes, and analysis of the expression of the cell adhesion protein, CD18, revealed an increase in the CD4^+^ and CD8^+^ subpopulations. CD18 is an integrin that is expressed by activated T lymphocytes in an antigen-specific manner and replaces the selectin receptor that is constitutively found in virgin cells that are restricted to secondary lymphoid organs. Thus, increased expression of this molecule could suggest a potential increase in the migration of subpopulations of T lymphocytes, mainly CD4^+^ T lymphocytes, toward the site of the inflammatory lesion, as indicated by the decline in the percentage of CD4^+^ T lymphocytes in the peripheral blood of patients. Yeh et al. (31) have demonstrated that people undergoing a regular 12-week program of music aerobic exercise, at a moderate level of physical activity, present changes in leukocyte distribution, lymphocyte subsets, and lymphocyte polarization. The participants also exhibited an increase in the frequency of CD4^+^CD25^+^ T cells associated with Treg polarization. Although we did not evaluate the functional phenotype of the CD4^+^ cells in this study, one can speculate that physical exercise of moderate intensity, such as the one employed here, can provided immunoregulation if cells with a regulatory phenotype are recruited to the site of inflammation. These cells may be able to influence the immune status of the patient and provide control of local inflammation, consequently resulting in clinical improvement.

The lymphocytopenia observed in the present study occurred only in the CD4^+^ subpopulation, with no change in CD8^+^ cells. Witard et al. (22) observed a reduction in CD8^+^ cells in response to intense exercise, demonstrating that the lymphocyte profile is highly sensitive to exercise and is largely driven by CD8^+^ T cells. However, different from our study, Witard et al. (22) examined young adults and the acute effect of exercise. Recently, Brown et al. (32) highlighted the importance of gender and training status for the redistribution of senescent and naive T lymphocytes in response to exercise. Moreover, Pereira et al. (33) demonstrated that the apoptosis and migration of CD4^+^ and CD8^+^ lymphocytes remain elevated 24 h after acute resistance training and that the cell count did not change at 2 or 24 h after exercise. CD8^+^ lymphocytes presented a higher responsiveness than CD4^+^ to a session of exercise with regard to apoptosis and migration. However, the sample was composed of twelve healthy, untrained, young individuals (mean age, 20.7 years), and the study focused on the effects of acute resistance training.

A possible explanation for the observation of lymphocytopenia after aerobic exercise is the movement of cells from the circulation (migration) and, most likely, from the involved joint. However, this hypothesis was not evaluated in the present study. Cells migrate to and from the lymphoid pools to maintain the homeostasis of immunity (33), and they migrate to and from inflamed joints to control the local inflammatory process.

It is important to emphasize the specificity of the sample of this study, which was composed of older women with an inflammatory joint disease. Studies have been conducted to determine the effect of a single session of exercise and of a training program, but some authors suggest that there is no interaction between acute exercise and training in the elderly (34). This phenomenon remains unclear in the studies specifically examining the effect of acute stress in the elderly population, and some studies have shown no changes in the recruitment of T-CD8^+^ lymphocytes. Thus, adaptations resulting from training cannot be accounted for by the sum of the results of each exercise session.

Another novel feature of this study was the increased CD18 expression on the surface of T lymphocytes (CD8^+^ and CD4^+^), as certain T-cell subsets were more likely to migrate. Only in the study by Pereira et al. (33) the effect of acute training on fluorescence intensity was examined. Similar to their study, the exercise training program described in this report activated more cells that may be related to the immune system's role in regaining control of the low-grade inflammation that is chronically observed in kOA. Moreover, as recently described, these active cell populations are more likely to exert a modulatory effect on inflammation and function to maintain the relative number of T cells (35). This capacity for the local modulation of inflammation may be associated with increased membrane expression of markers involved in adhesion to vessels and, therefore, greater migration to the target tissue.

Dorshkind et al. (36) reported the lower expression of CD28 on the surface of certain immune cells during the process of immunosenescence causing an inappropriate immune response, such as in the case of the reaction to vaccination. Thus, aerobic exercise training could promote an increase in percentage of CD8 T helper cells, resulting in a lower risk of infection and inflammation. In subjects with kOA, these changes in the immune response were not observed; however, it is important to emphasize that the positive immunomodulatory effect may be related to the magnitude of activation, because there was improvement in all of the clinical and functional parameters of the subjects after an aerobic training program.

It is worth noting that the present study sample was characterized as overweight, and after the intervention period, there was no change in body mass index. Because the load of body weight on the affected limb remained similar throughout the study, it is believed that the improvement in several domains of quality of life as well as the immunological changes were related to the training program and not the reduction of the weight overload on the affected limb. Another possibility to explain this positive effect on the self-perceived quality of life and immune parameters could be that the exercise training directly affected the adipose tissue, which has immunological effects. Nevertheless, the consistency of the present results for all patients is enough to support our conclusions. Furthermore, the results of this study provide subsidies for understanding the immunomodulatory effect of exercise in elderly with kOA.

There are limitations to interpreting the results of this longitudinal study. Therefore, the present results need to be confirmed in a randomly selected, larger sample of patients with stratification of the disease by phenotypes, and inclusion of males and a control group. We cannot exclude the possibility that the phenomenon observed with the immunological parameters in the patients with kOA in this study was an epiphenomenon reflecting some unknown mechanisms.

In conclusion, the results of the present study demonstrated that a walking training program (three times a week for 12 weeks) with a progressive and controlled load increase provided a significant improvement in all evaluated aspects of quality of life and in the physical performance of elderly women with osteoarthritis of the knee. Furthermore, this intervention also resulted in the trafficking and activation of leukocytes, which may be related to the immunomodulatory effect. Therefore, this study provides new insights showing that a simple training protocol composed of walking is sufficient to activate immune markers of activated lymphocytes. Future studies should be designed to investigate the specific mechanism, including joint analyses.
